# High Female Mortality Resulting in Herd Collapse in Free-Ranging Domesticated Reindeer (*Rangifer tarandus tarandus*) in Sweden

**DOI:** 10.1371/journal.pone.0111509

**Published:** 2014-10-30

**Authors:** Birgitta Åhman, Kristin Svensson, Lars Rönnegård

**Affiliations:** 1 Department of Animal Nutrition and Management, Swedish University of Agricultural Sciences, Uppsala, Sweden; 2 School of Technology & Business Studies, Dalarna University, Falun, Sweden; Institut Pluridisciplinaire Hubert Curien, France

## Abstract

Reindeer herding in Sweden is a form of pastoralism practised by the indigenous Sámi population. The economy is mainly based on meat production. Herd size is generally regulated by harvest in order not to overuse grazing ranges and keep a productive herd. Nonetheless, herd growth and room for harvest is currently small in many areas. Negative herd growth and low harvest rate were observed in one of two herds in a reindeer herding community in Central Sweden. The herds (A and B) used the same ranges from April until the autumn gathering in October–December, but were separated on different ranges over winter. Analyses of capture-recapture for 723 adult female reindeer over five years (2007–2012) revealed high annual losses (7.1% and 18.4%, for herd A and B respectively). A continuing decline in the total reindeer number in herd B demonstrated an inability to maintain the herd size in spite of a very small harvest. An estimated breakpoint for when herd size cannot be kept stable confirmed that the observed female mortality rate in herd B represented a state of herd collapse. Lower calving success in herd B compared to A indicated differences in winter foraging conditions. However, we found only minor differences in animal body condition between the herds in autumn. We found no evidence that a lower autumn body mass generally increased the risk for a female of dying from one autumn to the next. We conclude that the prime driver of the on-going collapse of herd B is not high animal density or poor body condition. Accidents or disease seem unlikely as major causes of mortality. Predation, primarily by lynx and wolverine, appears to be the most plausible reason for the high female mortality and state of collapse in the studied reindeer herding community.

## Introduction

Reindeer (*Rangifer tarandus tarandus*) herding in Sweden is a form of pastoralism practised by the indigenous Sámi population. The economy of reindeer husbandry is mainly based on meat production. Although the animals are domesticated (often referred to as “semi-domesticated”) and occasionally herded, they mostly graze freely on natural ranges.

Reindeer densities are normally regulated by harvest (slaughter) and intentionally kept below the ecological carrying capacity to enable sufficient harvest [1] (see Text S1 for details). Body condition is therefore mostly maintained at a relatively high level. Winter survival is enhanced by providing the reindeer with supplementary feed if needed to prevent possible malnutrition caused by adverse snow conditions (e.g. thick hard snow or ice). Female body condition may nevertheless affect calving success and early calf survival [2], and thereby the herd growth (and possibility for harvest), but is not expected to be the ultimate driver of population size.

In spite of seemingly good management and nutritional conditions, the harvest rate (harvest in relation to herd size) in Sweden is presently low and variable. Over the last decade, reindeer numbers have varied between 230 and 261 thousand animals in the winter herd (after the main harvest in autumn, but before new calves are born in the spring). The annual harvest has been 48–75 thousand reindeer per year, giving a meat production of 5.5 to 7.8 kg per live reindeer in the winter herd (statistics from the Sámi Parliament in Sweden, September 2013). A few reindeer communities, however, demonstrate the capacity to produce considerably more (a yearly production of 10–15 kg per live reindeer), which is closer to the production (commonly 10–20 kg per live reindeer) reported for Northern Finland [3].

**Figure 1:**
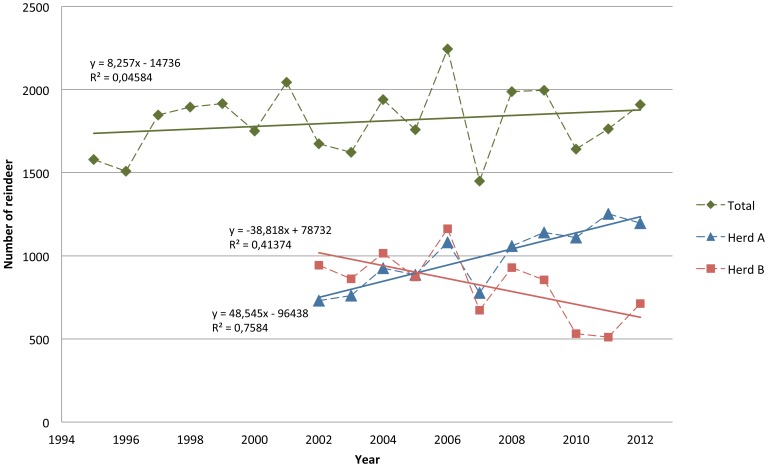
Number of reindeer in the winter herd according to yearly counts after harvest but before calving in spring from 1995 until 2012 (year referring to spring). Reindeer numbers are specified for herd A and B from 2002 and onwards, when the two herds began to be separated in winter (before this, all reindeer were kept in one large herd all year). The main owner of herd A (a young herder) built up his herd, explaining the gradual increase in the number of animals in herd A.

Declining harvest, without any subsequent increase in herd size, was observed in Njaarke reindeer herding community in central Sweden. A closer examination of the situation revealed an inability to keep up reindeer numbers in one of two sub-herds (herd B, Fig. 1), in spite of substantially reducing animal density in winter by moving almost half of the reindeer (herd A) to new winter ranges from 2001 and onwards, and a considerable drop in harvest from 2004 and onwards.

Negative herd growth may be due to high animal density or climatic factors, leading to poor body condition and subsequently low calf production and survival, effects of harvest on herd structure (not leaving a sufficient number of productive females), accidents, disease, predation, or a combination of these. In this paper, we present the results of research into female survival using capture and recapture records of individually marked female reindeer, collected over five years, together with results on body mass and calving success and official statistics on reindeer numbers and harvest, to examine possible reasons for the observed negative herd development and poor production in Njaarke reindeer herding community.

## Material and Methods

### Ethics Statement

**Figure 2:**
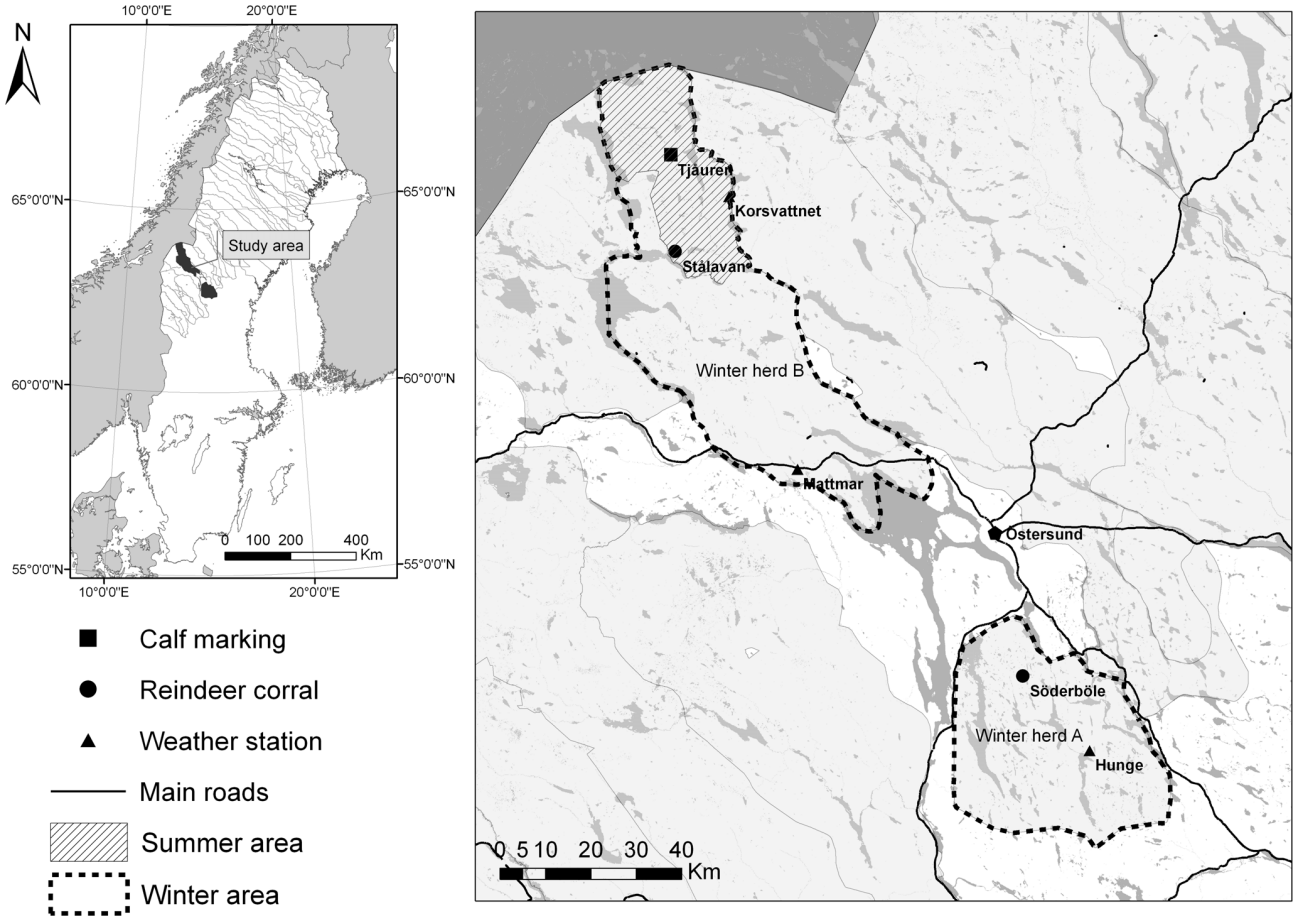
Map of study area with spring-summer-autumn ranges for the whole herding community and winter ranges for the two herds, and showing the main locations for reindeer gathering.

The investigated reindeer were all privately owned. Access to the animals and the area was provided by Njaarke reindeer herding community (indicated in Fig. 2). All fieldwork was made at facilities belonging to the herding community and in connection to routine handling of the animals. The only extra handling procedures, due to the research, was marking (using numbered collars on females and ear-tags on calves) and weighing of females and calves. This was approved and made in cooperation with the owners of the animals. No animals were sacrificed due to the project. The experimental design and handling of animals were approved by Umeå Ethical Committee for Animal Research (application A 35-07, A 76-10 and A 24-12). Official data on reindeer numbers and reindeer slaughter records was provided by the Sámi Parliament in Sweden (according to permission 2007-505). The use of herd-specific data was approved by the reindeer herding community (contact information is available at http://sametinget.se/8812).

### Study area

The study was carried out during 2007 to 2012 on privately owned reindeer in Njaarke reindeer herding community in the county of Jämtland, Sweden (62.56°–64.10°N, 12.89°–15.46°E; Fig. 2). The reindeer owners were organised in six enterprises, typically representing a family. All reindeer were marked with owner-specific cuts in their ears (according to common practice within reindeer husbandry). In summer, all reindeer were kept in one large herd of initially around 2000 animals, plus calves from the year. In this period, the reindeer grazed freely in sub-alpine and alpine habitats with a mean elevation 690 metres above sea level and a few high peaks up to 1250 m.a.s.l. This area is about 750 km^2^ and the animal density was around 2.5 animals per km^2^.

The reindeer were gathered for main harvest in October–December. At the same time, the remaining reindeer were counted and treated for parasites (injection with ivermectin). After this, the reindeer herd, about 75% of which were female reindeer over one year of age, was divided into two separate herds for winter grazing. The division was based on ownership of the individual reindeer. One herd (herd A, owned by two enterprises) was transported by lorry to an area with coniferous forest mixed with agricultural land, about 160 km south-west, whilst the other (herd B, owned by the other four enterprises) remained on forest land close to the mountain summer ranges. This division into two winter herds had been practiced from the autumn of 2001 and onwards. Before 2001, all reindeer grazed together on the winter ranges close to the mountains, which was later used only by herd B. Animal density on both winter ranges was about 0.5 reindeer per km^2^ at the start of the project in 2007. During the winter, the reindeer were regularly attended (by driving around the edge of the herd with snowmobiles several times a week). In early spring (late March or beginning of April), herd A was gathered and transported back to the mountain region where the animals again joined with herd B. The reindeer gradually moved from less to more elevated regions as spring advanced. Calving took place during May and the reindeer were gathered for calf marking in July.

According to data from the Swedish Meteorological and Hydrological Institute (SMHI) for the closest meteorological stations (indicated in Fig. 2), the winter temperature was about the same in both winter areas (the average temperatures for December to March varied from −4 to −11°C during the years 2007/08 to 2010/11, with minimum temperatures around −30°C). Total precipitation from December to March, in the area where herd A was located, varied between years from 113 mm to 134 mm. Substantially higher precipitation (210 mm to 339 mm) was reported at Korsvattnet, north of the winter area for herd B. This place is, however, known to have extremely high precipitation (because of westerly winds from the Norwegian Sea) which is not representative of the ranges generally used for herd B (the second weather station, at the southern border of area B, provided only temperature and not precipitation). Maps from SMHI still indicate somewhat higher winter precipitation in herd B's winter range than in that of herd A.

Eurasian lynx (*Lynx lynx*) and wolverine (*Gulo gulo*) were present in the area, both in the common summer ranges and in the winter ranges of both groups. The herders regard these two species to be the main predators on reindeer in this area. There is, however, no registration of reindeer that are found killed by predators in Sweden, since economic compensation for reindeer lost to predators is based on the presence of predators (yearly surveys) and not on found predator-killed reindeer [4]. These surveys, carried out by the County Administrative Board together with reindeer herders, showed that there were, in total, 3–5 lynx family groups and 1–2 wolverine dens (the investigated segments of these populations) within the land of the herding community during 2007–2011 (with no trend over time). In addition, three neighbouring communities from where predators may come hosted altogether 12–18 lynx family groups and 1–4 wolverine dens. Each family group of lynx or wolverine den is estimated to represent, on average, about 6 individuals [5], [6]. Brown bears (*Ursus arctos*) and eagles (*Aquila chrysaetos* and *Haliaeetus albicilla*) were present in unknown numbers. The herders claimed that eagles kill a number of calves each year, whereas bear predation was regarded as less of a problem by the herders in this area. There were also occasional visits by wolves (*Canis lupus*), but no reindeer kills by wolf were reported during the project period. Some protective culling of lynx has been allowed during the project period. In addition, there has been licensed hunting of lynx (in total, 49 lynx, including both licensed and protective hunting, were shot within the ranges of the herding community during 2007–2011). There was no protective culling of wolverine within the ranges of the herding community. However, on a few occasions, protective culling of single individuals of wolverine was allowed in neighbouring herding communities.

### Data collection and analyses

In total, 388 and 335 females from herd A and B, respectively, were originally marked with numbered collars in the project, either in the spring or in the autumn of 2007 (Table 1). Marked females represented 65% of the total number of females over one year of age in herd A and 66% of the females in herd B. Out of the marked females, 78 in herd A and 20 in herd B were eventually culled by the herders (mostly because of old age or poor fertility).

**Table 1:** Observations of marked females in spring (March–April), summer (July) and autumn (October–December) from 2007 until 2012.

	2007			2008			2009			2010			2011			2012		
	Spring	July	Aut.	Spring	July	Aut.	Spring	July	Aut.	Spring	July	Aut.	Spring	July	Aut.	Spring	July	Aut.
**Herd A**																		
Total marked^a^	300	300	388	367	367	366	353	352	352	346	346	346	330	326	326	310	310	310
Observed	300	208	325	230	263	274	241	176	171	218	221	213	196	218	195	176	170	82
Remain^b^	300	295	380	355	354	346	326	318	303	291	282	278	251	244	231	207	186	82
**Herd B**																		
Total marked^a^	-	-	335	-	335	335	-	329	329	-	325	325	-	323	322	-	315	315
Observed	-	-	335	-	214	253	-	89	117	-	140	130	-	115	103	-	76	33
Remain^b^	-	-	335	-	314	301	-	237	221	-	192	173	-	158	130	-	91	33

Results are presented as total number of marked females, observed females at the actual event and number of females known to still remain in the herd (observed at the actual event or later). Reindeer in herd B were all marked in the autumn of 2007 and were not gathered in the spring.

a Total marked = originally marked females minus those that have been deliberately removed from the herd (culled).

b Remain = Recaptured at the actual event or later – that is, verified as still remaining in the herd.

Recordings of the marked females in both herds took place at the autumn gathering in October–December and at calf marking in July. Females in herd A were also recorded in late March or at the beginning of April in connection with the lorry transport from winter to summer ranges. Calves of marked females were sexed, weighed and numbered with ear-tags at calf marking in July. Marked and recaptured reindeer (females and calves) were weighed at the autumn gatherings (see Text S2 for more details).

Reindeer counts (number of reindeer kept after harvest available from 1995 until 2012) and slaughter records (number of harvested reindeer from 1996/1997 until 2012/2013), divided on calves, females >1 year and males >1 year, and specified by owner, were used to estimate herd size and herd growth rate for the whole herding community and for the two herds A and B separately. This data was obtained from the Sámi Parliament in Sweden (retrieved in August 2013) (see Text S3 for details).

Annual herd growth rate before harvest *rG_t_* was calculated as:
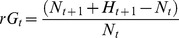
where

*N_t_* = winter herd size (counted number of reindeer after harvest) in year *t*

*H_t_* = harvested number reindeer in year *t*

Recruitment rate in autumn per female >1 year was calculated as the sum of harvested calves and counted calves after harvest, divided by the sum of harvested females >1 year and counted females after harvest.

Harvest rate was calculated for all reindeer (calves and adults together) and for calves separately, as harvested animals divided by the sum of harvested animals and counted animals after harvest.

Reindeer in Sweden are slaughtered at abattoirs and subject to the same regulations as other livestock. Slaughter records (including carcass mass and classification of fat content and muscle conformation according to the EUROP system applied in EU [7]) were used as indicator of overall reindeer body condition in the herding community [8]. Carcass fat and conformation (class variables) were transformed into quasi-normal distributions before calculations [8].

Capture-recapture analysis was used to estimate female survival over time in herds A and B. The Cormack-Jolly-Seber (CJS) model for live recaptures was chosen for the capture-recapture analysis [9], [10], [11]. The model uses two parameters: survival rate between encounter occasions (*φ*) and recapture rate for each encounter occasion (*p*). The animals reported as harvested were removed from the analysis after their last encounter occasion, usually in the autumn. The CJS model was fitted using the R package RMark, an R interface for the program MARK (version 7.1).

Herd A and herd B were separated in winter, and group effects in both survival and recapture were therefore assumed. An interaction effect between time and group, allowing the group effect to vary over time, was assumed when estimating survival rate. Both herds were captured on the same occasion in July and in the autumn, and hence no interaction effect was added to the recapture rate. Alternative models having year and herd as effects were assessed and the model with lowest AICc was chosen (see Text S4 for details). As herd B was not recaptured in the spring, the recapture rate for these occasions was set to zero for this herd. The survival rate for herd B between spring and the next encounter in July was accordingly fixed to a value of one. Using this method, the estimates were not biased by fewer recapture occasions for herd B.

Some survival estimates were close to the parameter boundary (close to 1.0). For herd A, the survival rates between spring and July in 2007 and 2008 were estimated to one. This was also the case for herd B's survival between July and autumn 2008. To avoid numerical problems in model fitting, these parameters were all set to a fixed value of one. The survival rate between the last two recaptures (spring to July 2012 and July to autumn 2012) was not identifiable by definition and therefore set to one.

In a separate analysis, we used logistic regression to test the effect of year, herd and autumn live body mass (BM) on the females' chance of survival until the next autumn. We also tested the effects of herd and year on female live BM and mass and classification of harvested reindeer, respectively (for live BM we also included sampling event within year in the model). The effect of year and herd on calving success for marked females (calf or no calf at calf marking in July) was tested using a logistic model. Logistic models were also used for testing the effects of year and herd on herd growth rate, recruitment rate in autumn and harvest rate. These analyses were carried out using JMP statistical software, version 9.0.2.

A reindeer herd having no, or very small, long-term growth can only persist for a few years because of the economic demands of the production system. It will lead to an irreversible decline (i.e. collapse) of the herd and there will be a break point where the mortality will be too large. A rough value of the mortality leading to this break point was computed by assuming 60% of the adult females (>2 years) have a calf surviving until its first autumn (as observed in the current study) and that old females (>10 years) were culled [12]. Furthermore, two different scenarios were considered. In the first “high relative calf survival” scenario, the survival of calves between their first two autumns was assumed to be 80% of the adults' survival (which might be the case in a situation where e.g. accidents would be a main cause of mortality), whereas the corresponding value for the second “low relative calf survival” scenario was 50% (a more likely scenario if poor nutrition [13] or predation [14] is the main cause of mortality). The adult mortality rate resulting in a unit dominant eigenvalue for the Leslie matrix [15] having the above parameters was defined as the maximum sustainable mortality.

## Results

### Herd size, herd growth and harvest

As illustrated by Fig. 1, the yearly reindeer counts for the whole reindeer herding community (herd A and B together) showed no significant trend from 1995 until 2012. Until 2001, the annual herd growth rate was on average 0.40%, which was balanced by a harvest of 501–715 reindeer per year, keeping the herd size stable (see Text S5 for more details).

After the winter of 2001/2002, when herd A started to use novel winter grounds, the annual herd growth rate before harvest was significantly higher (P<0.05) in herd A than in herd B (LSM = 0.335 and 0.213, respectively, s.e. = 0.035). Recruitment rate (calves per female >1 year) in autumn was on average 0.50, and did not differ between the two herds. The harvest rate of calves (per cent harvested of available calves before harvest) was also significantly larger (P<0.05) for herd A than for herd B (49.6% compared to 36.2%, s.e. = 3.3%). Harvest rate for all reindeer (calves and adults together) did not differ between the two herds. Herd growth, recruitment rate and harvest rate varied significantly between years, but there were no significant trends over time.

Reindeer numbers after harvest increased with on average 48±9 animals (mean±s.e.) in herd A, whilst there was a declining trend in herd B, corresponding to 38±15 reindeer less per year. The increase in reindeer numbers in herd A was deliberate, since the main reindeer owner (a young herder) was keeping many reindeer calves to build up his herd. Total reindeer numbers were persistently well below the maximum for the herding community (2700 reindeer), set by the authorities (the County Administrative Board of Jämtland).

### Female survival

**Figure 3:**
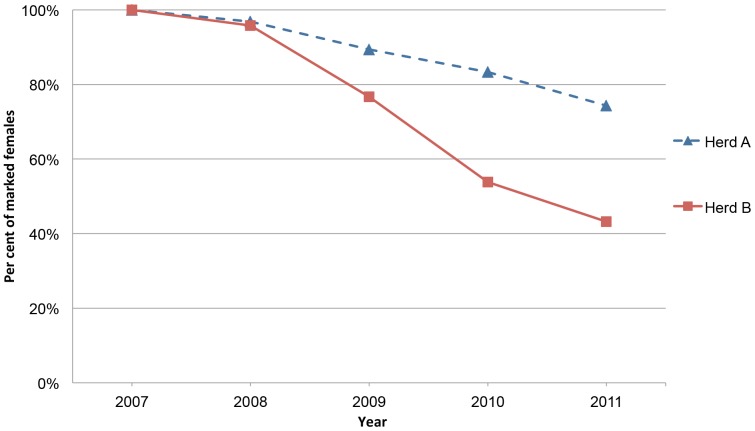
Accumulated survival rate of females in Herd A and B from autumn 2007 until autumn 2011, calculated using estimates from capture-recapture analysis shown in **Table 3** (showing also the confidence intervals for the estimates).

Out of the females originally marked in 2007, 231 in herd A and 130 in herd B were shown to still remain in the autumn of 2011, after four years (Table 1). Recapture rates for the different sampling occasions (Table 2) varied between 0.50 and 0.84 for herd A and between 0.38 and 0.76 for herd B, except for the last recapture in autumn 2012 when it was lower (0.39 and 0.28, respectively). The estimated yearly survival rate from one autumn to the next (based on survival rates shown in Table 3, with the last year excluded) was, on average, 92.9% for herd A, and considerably lower, 81.6%, for herd B. In the autumn of 2011, after four years, 74% of the females in herd A and only 43% of those in herd B, that had not been culled, were estimated to still be present in the herd (Fig. 3).

**Table 2:** Estimated recapture rate for females in herd A and B (estimate and 95% confidence interval) based on analysis of the capture-recapture of individually marked adult females.

	Herd A		Herd B	
Time	estimate	95% conf.int.	estimate	95% conf.int
July 2007	0.70	0.65–0.75	-	-
Autumn 2007	0.81	0.76–0.85	-	-
Spring 2008	0.65	0.60–0.69	-	-
July 2008	0.75	0.72–0.79	0.64	0.60–0.69
Autumn 2008	0.83	0.80–0.86	0.74	0.70–0.78
Spring 2009	0.73	0.68–0.78	0	-
July 2009	0.50	0.46–0.55	0.38	0.33–0.42
Autumn 2009	0.57	0.52–0.61	0.44	0.39–0.49
Spring 2010	0.74	0.69–0.79	0	-
July 2010	0.78	0.74–0.82	0.68	0.63–0.73
Autumn 2010	0.78	0.74–0.82	0.68	0.62–0.73
Spring 2011	0.78	0.72–0.83	0	-
July 2011	0.84	0.80–0.87	0.76	0.70–0.80
Autumn 2011	0.82	0.78–0.86	0.74	0.68–0.79
Spring 2012	0.83	0.77–0.88	0	-
July 2012	0.78	0.72–0.83	0.67	0.60–0.74
Autumn 2012	0.39	0.05–0.89	0.28	0.03–0.83

Most females in herd A were marked in spring 2007 and the rest in the autumn of 2007. Reindeer in herd B were all marked in the autumn of 2007 and were not gathered in the spring.

**Table 3:** Estimated survival of females in herd A and B from one sampling event to the next (estimate and 95% confidence interval), based on analysis of the capture-recapture of individually marked adult females.

	Herd A		Herd B	
Time	estimate	95% conf.int.	estimate	95% conf.int
July 2007	0.988	0.958–0.996		-
Autumn 2007	0.988	0.954–0.997		-
Spring 2008	0.989	0.965–0.997		-
July 2008	1.000	(fixed)	0.958	0.919–0.979
Autumn 2008	0.980	0.953–0.991	1.000	(fixed)
Spring 2009	0.985	0.947–0.996		-
July 2009	1.000	(fixed)	0.830	0.746–0.891
Autumn 2009	0.937	0.894–0.963	0.965	0.593–0.998
Spring 2010	0.975	0.922–0.992		-
July 2010	0.962	0.924–0.982	0.793	0.695–0.866
Autumn 2010	0.994	0.880–1.000	0.884	0.803–0.935
Spring 2011	0.949	0.907–0.973		-
July 2011	0.994	0.858–1.000	0.946	0.833–0.984
Autumn 2011	0.945	0.900–0.970	0.848	0.738–0.917
Spring 2012	0.977	0.907–0.995	0.868	-

Most females in herd A were marked in spring 2007 and the rest in the autumn of 2007. Reindeer in herd B were all marked in the autumn of 2007 and were not gathered in the spring. Dates refer to the end of the survival period.

Two scenarios, corresponding to a high and a low relative calf survival, respectively, were assessed to compute the level of adult mortality where a reindeer herd collapses. The maximum sustainable mortality was 17% for the high calf survival scenario and 7.5% for the low calf survival scenario (where poor grazing conditions or predation is expected to affect calves substantially more than adults).

### Body condition and calving success of marked females

The live body mass (BM) of the marked females varied from 50 to 93 kg at the autumn gathering. Eighty per cent of the observations were within the range 63–79 kg. Female BM varied year to year (Table 4), and also between sampling occasions within the year, but we found no general trend over time, except for significantly lower BM the first autumn (2007) compared to later years. There was a slight, but still statistically significant (P = 0.038), effect of herd on female BM, with, on average, 0.7 kg higher BM in herd A compared to herd B.

**Table 4:** Autumn live body mass of marked females (least square mean and standard error) in each of the observed years and overall for the two herds A and B.

	n	LSM	s.e.	sign.
**Year, month**				
2007, Nov	215	64.9	0.43	a
2008, Oct	212	72.2	0.39	bc
2008, Nov	62	69.3	0.75	d
2009, Nov	155	71.1	0.46	cd
2009, Dec	112	70.7	0.54	cd
2010, Nov	289	71.8	0.34	bcd
2011, Nov	180	73.2	0.43	b
2011, Dec	87	70.9	0.62	cd
**Herd**				
Herd A	830	70.9	0.23	a
Herd B	482	70.2	0.28	b

Values with the same letter within effect (year and group, respectively) are not significantly different (sign. level is set at P<0.05).

Due to technical problems with the scales, not all females were weighed on all gathering occasions. We could, therefore, not include the autumn BM in the capture-recapture analysis (too much missing data). Nevertheless, based on data from the females that were weighed, we found no significant effect of individual female BM in one autumn on the chance of her survival until the following autumn (recaptured the following autumn or later) when we analysed the entire dataset using a statistical model with herd, year and autumn BM as fixed effects. When testing the effect of female autumn BM on the chance of recapture for each herd and year separately, we found no effect for herd A in any of the years. For herd B, we found a small effect of BM in the autumn of 2009 (P = 0.035) on loss by the autumn of 2010, but no effects in the other years (Table 5).

**Table 5:** Autumn live body mass of females in the two herds that were recaptured or not recaptured, respectively, the following autumn or later (number of weighed females, mean and standard error).

	Recaptured			Not recaptured			sign.
from – to	n	mean	s.e	n	mean	s.e	P-value
**Herd A**							
2007–2008	206	65.4	0.4	9	63	1.9	not sign.
2008–2009	93	71.2	0.6	9	73.2	2.1	not sign.
2009–2010	142	71.8	0.4	12	72.3	1.4	not sign.
2010–2011	160	72.2	0.5	16	72.5	2	not sign.
**Herd B**							
2008–2009	125	71.6	0.6	47	71.5	0.7	not sign.
2009–2010	80	70.7	0.5	31	68.7	0.9	*P = 0.035
2010–2011	88	71.6	0.6	26	70.5	1	not sign.

P value for significant difference between recaptured and not recaptured. Females in herd B were not weighed in autumn 2007.

Annual calving success among marked females was significantly affected by both year (P<0.0001) and herd (P = 0.0003), with a mean calving success (marked females with calf) in July of 68% for herd A (58–76% for single years) and 57% for herd B (46–64% for single years). There was some variation between years in the body mass of marked calves, both in summer and autumn, but no general trend over the whole project period. Calves from herd A weighed slightly (less than 1 kg) more than those from herd B, both at calf marking in July and at the gathering in the autumn (LSM ± s.e. was 22.9±0.10 kg for herd A compared to 22.1±0.16 kg for herd B in July, and 41.7±0.28 kg compared to 41.1±0.37 kg in autumn).

### Body condition of harvested reindeer

The average carcass mass of slaughtered calves was generally lower in the autumn of 2007 (19.2 kg) compared to the following years (between 20.2 and 20.5 kg). A corresponding difference was found for slaughtered females (29.5 kg in 2007, compared to 32.6–35.2 kg in the following years). The carcass fat classification was significantly lower for both calves and females in 2007 compared to the following years, whilst we found no pattern with regards to carcass conformation (amount of muscle). We found no significant differences between herds A and B in carcass mass, fat or conformation during the project period (2007–2012), either for calves or females.

## Discussion

### Reindeer mortality and herd dynamics

Capture-recapture analysis reveals a substantial loss of adult females in the reindeer herding community studied and this affected both herds. The situation for herd B was, however, detrimental, with more than twice the mortality rate for females compared to herd A. Based on the total number of females over 1.5 years of age in the respective herd (data from the Sámi Parliament in Sweden), the observed annual loss of 7.1% in herd A corresponded to an average loss of 55 females per year, whereas the loss of 18.4% in herd B corresponded to, on average, 100 females per year (however, a declining number as the total number of reindeer in herd B declined). Adult males would be expected to be lost at roughly the same rate as females (irrespective of cause), whilst calves would probably be lost at a higher rate. The same risk of mortality for reindeer older than 1.5 years as for adult females, and a risk twice as high for young reindeer (0.5–1.5 years old), correspond to additional annual losses of about 50 reindeer (males and calves) in herd A and 100 in herd B. In addition, the loss of an adult female creates a gap in calf production until this female has been replaced with a new young female. It is, thus, not surprising that the observed loss of females in herd B caused an inability to maintain reindeer numbers (in spite of a very low harvest) and a subsequent decline in herd size, demonstrating a continuing decline (collapse) of the herd.

The estimated breakpoint for when herd size cannot be kept stable confirms that the observed female mortality rate in herd B represents a state of herd collapse, even in the case of a high calf survival (80% of adult survival). Using a more probable scenario (consistent with the discussion above), with half the survival for calves compared to adults, the situation for herd B (18% female mortality) greatly exceeds the estimated breakpoint for herd collapse (7.5%).

Occasional reductions in reindeer numbers are common and have often been associated with harsh winters, resulting in greater than usual winter mortality rates, especially among young animals, followed by impaired calf production the next summer e.g. [16], [17]. Icing of ground vegetation is often suggested as a main reason e.g. [18], although winter precipitation, which is easier to monitor in a consistent way, seems to be a more solid explanatory factor for extensive mortality and reductions in reindeer numbers [19]. It should be noted that the year to year variation in the counted number of reindeer (as shown in Fig. 1) does not necessarily reflect true changes in herd size, but may be due to varying success in gathering and counting the herd. In addition, variations in the size of the winter herd are partly an effect of a variation in harvest rate between years.

Persistent declines in reindeer numbers over many years have usually followed a peak in animal numbers, and been linked to high animal densities and overuse of pastures, especially winter/lichen ranges e.g. [20], [21], [22], [23]. Variations in climate are proposed as an additional reason for such variations in numbers [16], [24], [25]. Population reduction, primarily caused by depleted food resources, may be accelerated by other factors such as severe winter weather, excessive harvest or predation.

Situations of herd collapse are unusual nowadays in domesticated reindeer but have been described previously, e.g. a population crash in Finland in 1973 [26]. It was reported that, after this crash, supplementary feeding of reindeer became common in Finland to prevent future crashes. There are, nevertheless, recent examples of substantial drops in herd size, associated with high reindeer densities and low calf production in Finnmark, Norway [27]. As discussed by Pape and Loeffler [28] and Ulvevadet and Hausner [29], this cannot be explained solely by ecological mechanisms, but is a combined effect of socio-economic factors, management decisions and ecology.

### Animal density and body condition

Reindeer density in summer in the herding community studied was close to the average for reindeer herding in Sweden [30], whilst winter density was lower than the average. According to available records, animal numbers have been kept substantially below the allowed 2700 reindeer since 1996 and show no general trend over time (as shown in Fig. 1). Historical reindeer numbers are however unclear. In a development plan for the herding community from the County Administrative Board of Jämtland [31], it was reported that the number of reindeer counted in 1989 was 2181, thus slightly greater than the numbers reported for the last 18 years.

Animal density in winter was substantially reduced (almost halved) after 2001 when the two herds A and B where separated in autumn, and herd A started to use novel winter ranges. In spite of this, and the gradual reduction in harvest, reindeer numbers declined in herd B. The continued reduction of reindeer density for herd B in winter did not improve survival over time, suggesting that the high female mortality in this herd was not an effect of high density on the winter ranges.

Slightly lower autumn body mass and calving success among the marked females in herd B compared to those in herd A imply better winter conditions for herd A than B. This might be attributed to differences in winter foraging conditions (probably more snow in the winter area of herd B compared to that of herd A) and, thus, female fitness in spring, although we did not have any opportunity to directly measure such an effect. The difference between the two herds was, however, not confirmed by the observed carcass weights and classification of harvested reindeer or recruitment rate in autumn. There may have been a difference in age structure among females between the two herds, affecting both average body mass and calving success. High female mortality forces the reindeer herders to keep almost all female calves, creating a herd with a large proportion of young females, which are not expected to produce as many and as heavy calves as the older ones [32]. It might also be that the herders are more reluctant to culling old females when many adult females are lost.

Even if different winter grazing conditions may explain differences in calf production between the two herds, it is not a plausible explanation of the more than double female mortality in herd B compared to A. As concluded by Gaillard et al. [33], adult female survival in herbivores generally shows little variation and little dependence on animal density. Long before adult animals start to die because of inadequate access to food, their fecundity is expected to drop dramatically. This is in line with findings on wild reindeer in Norway [20], [34], [35], stating that that density-dependent limitation of food during winter did not influence adult survival, even though calf survival the following summer was significantly affected. If poor nutrition had been a major reason for the differences in female mortality between the two herds, we would have expected a more marked effect of herd on calving success in July and an effect on recruitment rate in autumn, which we did not find. Furthermore, females with lower body mass in autumn would be expected to have a higher risk of dying due to poor grazing conditions during winter than heavier females, which also disagrees with our results.

### Possible role of predation

Predation has been shown to be a major cause of reindeer mortality in Sweden [36], as well as in Norway [37] and Finland [38], [39], [40]. Kill rates for lynx vary depending on season, sex and social status of the lynx, but were shown to be around 4–8 reindeer per month in winter within areas with large numbers of reindeer [41]. This is in line with previous findings [42] revealing kill rates for female lynx with kittens at six reindeer per month in winter. Depending on sex and age, lynx select different categories of reindeer[14]. Lynx generally prefer calves, but male lynx switch to selecting a larger proportion of adult reindeer in winter. Kill rates for wolverine are unclear, since they are opportunistic predators and also scavenge on prey killed by other predators [43]. According to the latter study, kill rates of lynx on reindeer were nine times greater than that of wolverine in areas where both predators were present. However, when snow conditions are favourable, wolverines may kill many reindeer at one single event [36], [44]. It has been shown by Hobbs et al. [45] that both lynx and wolverine reduce the harvest of reindeer in Sweden, with almost 100 reindeer per family groups of lynx (95% confidence interval = 31–155 reindeer) or wolverine (20–160 reindeer), even when climate and reindeer density were taken into consideration in the statistical models.

Actual kills of reindeer by predators have not been registered in the study area. Dead reindeer are regularly found, but these represent only a fraction of those that are lost, and the rate and cause of mortality can therefore not be quantified based on these findings. As earlier described, reindeer herders are compensated for predation losses based on the amount of predators (mainly lynx and wolverine) present within a reindeer herding community. The system was established in 1996 because of the large errors involved in basing the compensation on found reindeer, and as a means to increase the reindeer herders' acceptance to the presence of predators. It has however had the effect that dead reindeer are not actively searched for and there are no statistics on found reindeer killed by predators.

Both lynx and wolverine are present within the ranges of the herding community studied. Using a conversion factor of 6 [5], [6], the number of wolverine dens (1–2) and lynx family groups (3–6) would correspond to 6–12 wolverines and 18–30 lynx individuals. It is obvious that this is not a fixed relationship and that the number of individuals represented by a den or family group will vary both geographically and over time. Herders of herd B claimed that there were many adult male lynx within their winter ranges, and that these animals were responsible for most kills of adult reindeer. Assuming that each lynx family group corresponds to 4.5 independent adult animals in winter [5], with a kill rate of a minimum of 4 reindeer per month [41] during November until April, two lynx family groups would be enough to result in the annual loss of a total of the 200 reindeer suggested above for herd B (not counting mortality of calves in summer).

Even at very low reindeer densities, family groups of lynx (females with kittens) may effectively prey on the few that are present [41]. With the same number of lynx, predation rate is thus actually expected to increase as the number of reindeer decline. This is consistent with our observation that the decline in herd B seems to have started after herd A was removed from the previously common winter ranges.

Although there were no documented differences in the number of predator family groups in the winter area of herd A compared to that of herd B, the former herd lost far fewer reindeer. As stated above, the ratio between actual number of individuals and the number of family groups of lynx or wolverine is not constant [5], [6], and the kill rates of lynx on reindeer may vary substantially depending on season, reindeer density and availability of alternative prey [43]. According to the owners of herd A, they found reindeer killed by lynx in their winter area only occasionally. The herders' explanation was that a high density of roe deer (*Capreolus capreolus*), a preferred prey for lynx [46], [47], usually provided the predators with enough food such that reindeer was therefore not their main prey in this area. Hunting statistics from the Swedish hunters association [48] confirms that there were more roe deer in the area of herd A (Revsund hunting district) than in that of herd B (Krokom hunting district). Large differences in predation rates, in spite of similar numbers of family groups of lynx and wolverine, is in accordance with the large variation found by Hobbs et al. [45] on the effects of lynx and wolverine on reindeer production. Even if the situation for herd A was considerably better than for herd B, the observed annual loss of 7.1% of prime aged females evidently has negative affects on herd growth and room for harvest.

### Other possible causes of mortality

Reindeer killed by train or cars are normally reported, since reindeer owners are refunded for these losses. During the project period there were no train accidents, and very few reindeer were killed by cars (none of these were marked females). The topography of the land is not such that accidents due to falling, drowning or other mortal injury should be common (the highest elevated areas are not used by the reindeer in winter). No outbreaks of disease are known and the reindeer are regularly treated against parasites. Earlier research indicates that disease prevalence is generally low among reindeer and caribou [36], [49], [50]. There is thus little reason to believe that illness would be a major cause of mortality in the studied herd.

## Conclusions

We conclude that there is an on-going collapse in one of the two reindeer herds studied and that the prime driver of this collapse is not poor body condition of the animals. Accidents or disease seems unlikely as major causes of the observed female mortality. Predation, primarily by lynx and wolverine, appears to be the most plausible reason for the high female mortality and state of collapse in the studied reindeer herding community. To keep the herd stable and allow for a reasonable harvest after recovery, reindeer mortality has to be kept at a substantially lower level than has been the case in this herding community over the last decade. The project demonstrates the importance of records of identifiable individual animals in order to obtain good estimates of reindeer mortality and be able to capture (or exclude) causes of this mortality.

## Supporting Information

Text S1 **Organisation of reindeer herding in Sweden.** This text provides details on ownership, access to land and regulation of reindeer herd size in Sweden.
(PDF)

Text S2 **Registration and weighing of females and calves.** This text describes how reindeer were gathered, registered and weighed in autumn and spring and for calf marking in July.
(PDF)

Text S3 **Reindeer counts and slaughter records.** This text describes details on annual reindeer counts and slaughter records that are reported to the Sámi Parliament.
(PDF)

Text S4 **Survival model comparisons.** This text describes comparison of four different models for estimating survival (*φ*) and recapture (*p*).
(PDF)

Text S5 **Herd growth, recruitment and harvest.** This text gives details on annual changes in herd size, recruitment of new calves and harvest from 1996 until 2001 for the whole (combined) herd, and from 2002 until 2011 for herd A and B separately.
(PDF)
