# Supplementary material for: High Female Mortality Resulting in Herd Collapse in Free-Ranging Domesticated Reindeer (Rangifer tarandus tarandus) in Sweden
Source: PLoS One. 2014 Oct 30;9(10):e111509. doi: 10.1371/journal.pone.0111509 (PMC4214728; doi:10.1371/journal.pone.0111509)
Supplement: Text S1 — Organisation of reindeer herding in Sweden. This text provides details on ownership, access to land and regulation of reindeer herd size in Sweden. (PDF) [file pone.0111509.s001.pdf]

Text S1. ORGANISATION OF REINDEER HERDING IN SWEDEN

Reindeer herding in Sweden is organized in 51 herding communities ("sameby" in Swedish), each consisting of several reindeer owners who herd their privately owned reindeer together. Each herding community has access to a defined area of land for reindeer grazing, and winter ranges are often further divided among the individual reindeer owners. The economic goal is to keep an optimal number of animals (primarily productive females) in relation to food resources, thereby maximizing growth, reproduction and possible harvest on the available land. Reindeer densities are therefore kept below the ecological carrying capacity of the system [1], although there might be difficulties in deciding or agreeing on targets for reindeer numbers for the respective herding communities, as discussed by Olofsson [2]. Local government regulations regarding "maximum number of reindeer" for single herding communities also set a limit on the number of animals. The herd growth is further optimized by harvesting a majority of the male calves, usually keeping just enough males for mating, and removing old and infertile females.

References

1. Scoones I (1993) Economic and ecological carrying capacity: applications to pastoral systems in Zimbabwe, in *Economics and ecology: new frontiers and sustainable development.*, Barbier EB, Ed. Chapman & Hall Ltd: London, UK. p. 96-117
2. Olofsson A (2011) *Towards adaptive management of reindeer grazing resources*. PhD thesis. Acta Universitatis Agriculturae Sueciae. Vol. 2011:16. 161 pages.
