# Supplementary material for: High Female Mortality Resulting in Herd Collapse in Free-Ranging Domesticated Reindeer (Rangifer tarandus tarandus) in Sweden
Source: PLoS One. 2014 Oct 30;9(10):e111509. doi: 10.1371/journal.pone.0111509 (PMC4214728; doi:10.1371/journal.pone.0111509)
Supplement: Text S2 — Registration and weighing of females and calves. This text describes how reindeer were gathered, registered and weighed in autumn and spring and for calf marking in July. (PDF) [file pone.0111509.s002.pdf]

Text S2 - REGISTRATION AND WEIGHING OF FEMALES AND CALVES

Autumn gathering

During the project period, there were one or two large gatherings each autumn (October-December) in Njaarke reindeer herding cooperative. Some small gatherings were made later to collect reindeer flocks that had been missed at earlier gatherings. The reindeer were first herded into a large corral. Thereafter small groups of reindeer (around 50 individuals) were taken into a small enclosure where each animal was captured by hand and identified on owner based on a traditional mark cut into the ears for the annual counting. After selecting animals for harvest, the remaining reindeer were treated against parasites and individually marked reindeer for the project were weighed. The reindeer were then transferred to different enclosures based on winter herd (A or B), before they were either transported by lorry to winter ranges further south (herd A) or herded to neighbouring winter grazing ranges (herd B).

Spring gathering (herd A only)

Herd A was gathered in the spring (late March or beginning of April) for transport back to the common grounds for herd A and B. The reindeer where herded into a large corral from where they were taken in smaller groups through a corridor, where the individually marked reindeer were registered and weighed, before they were loaded onto a lorry for transport.

Calf marking

All reindeer were gathered in July for calf marking. They were first herded into a large corral. From this they were taken in groups of around 500 reindeer (not counting the new calves) into a smaller enclosure. The calves, that usually follow the dam, were identified by owner based on the earmark of the dam, and captured using a so-called vimpa (a snare on a long stick). Calves of individually marked females for the project were also identified by ID of its dam. All individually marked females that were present in the enclosure were noted on a list.

General note

It is not possible to gather 100% of the animals at each of the above events since they roam over large areas (in the order of hundreds of km^2). We might also have missed a few animals, not reported by the herders, from small extra gatherings in autumn and spring that were made to capture animals that had not been found at the main gatherings.
