# Supplementary material for: High Female Mortality Resulting in Herd Collapse in Free-Ranging Domesticated Reindeer (Rangifer tarandus tarandus) in Sweden
Source: PLoS One. 2014 Oct 30;9(10):e111509. doi: 10.1371/journal.pone.0111509 (PMC4214728; doi:10.1371/journal.pone.0111509)
Supplement: Text S3 — Reindeer counts and slaughter records. This text describes details on annual reindeer counts and slaughter records that are reported to the Sámi Parliament. (PDF) [file pone.0111509.s003.pdf]

Text S3 - REINDEER COUNTS AND SLAUGHTER RECORDS

Annual counts of reindeer (specified by owner and divided on calves, females >1 year and males >1 year) are made by the herders at autumn and winter gatherings in all reindeer herding communities in Sweden, and reported to the Sami Parliament in the spring. At these gatherings reindeer are also selected for harvest (slaughter) and the main herd is usually divided into smaller winter herds (based on owner). The reported counts include only the reindeer kept over winter, and thus not those that are harvested. All counted reindeer are marked with colour on the coat to avoid repeated counting of the same animal (this colour will be lost until the next year, due to shedding of the winter coat). Reindeer counts are regularly controlled by the County Administrative Board of the relevant county (Jämtland, Västerbotten or Norrbotten).

In Sweden the slaughter of reindeer is subject to the same EU regulations as slaughter of other livestock. Thus the majority of reindeer are slaughtered at commercial abattoirs. Slaughtered reindeer (specified by owner and divided on calves, females >1 year and males >1 year) are reported by the slaughter companies to the Sami Parliament. Some reindeer for household consumption are slaughtered by the herders themselves and thus not reported. Subsidies are paid to the herders based on slaughtered reindeer reported to the Sami Parliament, which reduces the motivation for own slaughter. According to own observations and information from the herders, very few reindeer in Njaarke are slaughtered outside the abattoirs. In our calculations of herd growth and harvest rate we have therefore used only the harvest reported to the Sami Parliament.
