# Supplementary material for: High Female Mortality Resulting in Herd Collapse in Free-Ranging Domesticated Reindeer (Rangifer tarandus tarandus) in Sweden
Source: PLoS One. 2014 Oct 30;9(10):e111509. doi: 10.1371/journal.pone.0111509 (PMC4214728; doi:10.1371/journal.pone.0111509)
Supplement: Text S4 — Survival model comparisons. This text describes comparison of four different models for estimating survival (φ) and recapture (p). (PDF) [file pone.0111509.s004.pdf]

Text S4 - SURVIVAL MODEL COMPARISONS

Capturing of animals is performed in collaboration between the owners of Herd A and B. Hence, having no interaction effect between herds for recapture is the most biologically founded model, which was also supported by the comparison of AICc-criterion for several alternative models (Model 1 in Table S4.1).

Candidate models evaluated the possibilities to let both survival (φ) and recapture (p) vary over time and within herds (i.e. there is an interaction effect between time and herd, Model 2), a constant group effect for φ and/or p (Model 3) and also a model without any herd-effect for p (Model 4).

The survival estimates varied slightly between the different models but the general pattern was still the same (see Fig. S4.1, S4.2 and S4.3 below).

Table S4.1. Comparisons of different models for survival (φ) and recapture (p)

**Model**	**Number of parameters**	**AICc**	**ΔAICc**
Model 1<br>$\varphi(time + herd + time * herd)$<br>$p(time + herd)$	40	23633.5	
Model 2<br>$\varphi(time + herd + time * herd)$<br>$p(time + herd + time * herd)$	48	23639.5	6
Model 3<br>$\varphi(time + herd)$<br>$p(time + herd)$	34	23660.8	27.3
Model 4<br>$\varphi(time + herd)$<br>$p(time)$	32	23709.9	76.4

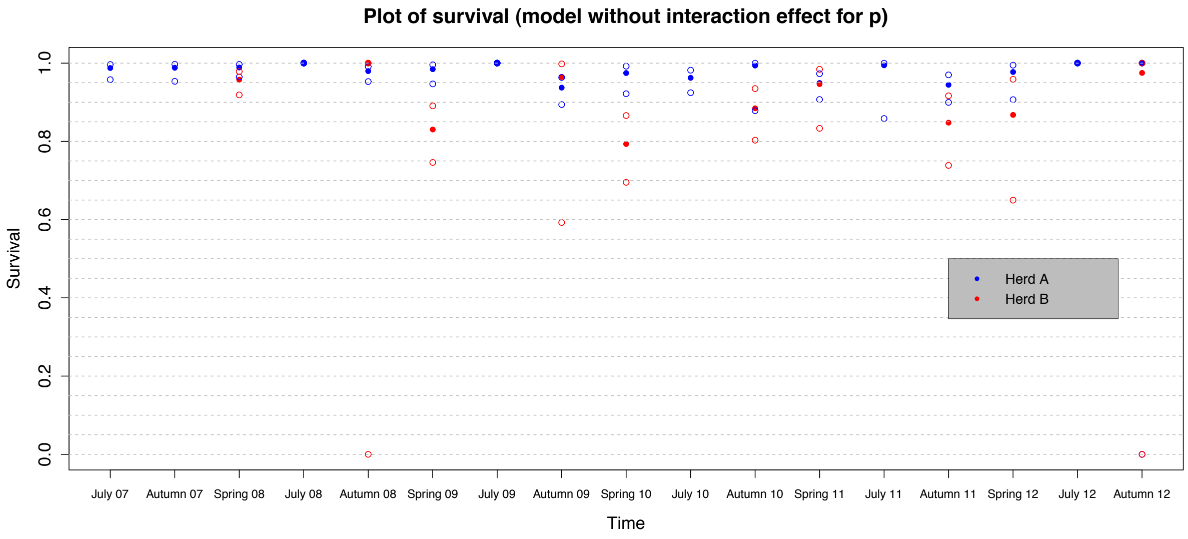

Fig S4.1. Survival estimates (filled circles) with lower and upper ends of 95% confidence intervals (open circles) for Model 1 in Table S4.1. Non-identifiable estimates have confidence intervals from 0 to 1.

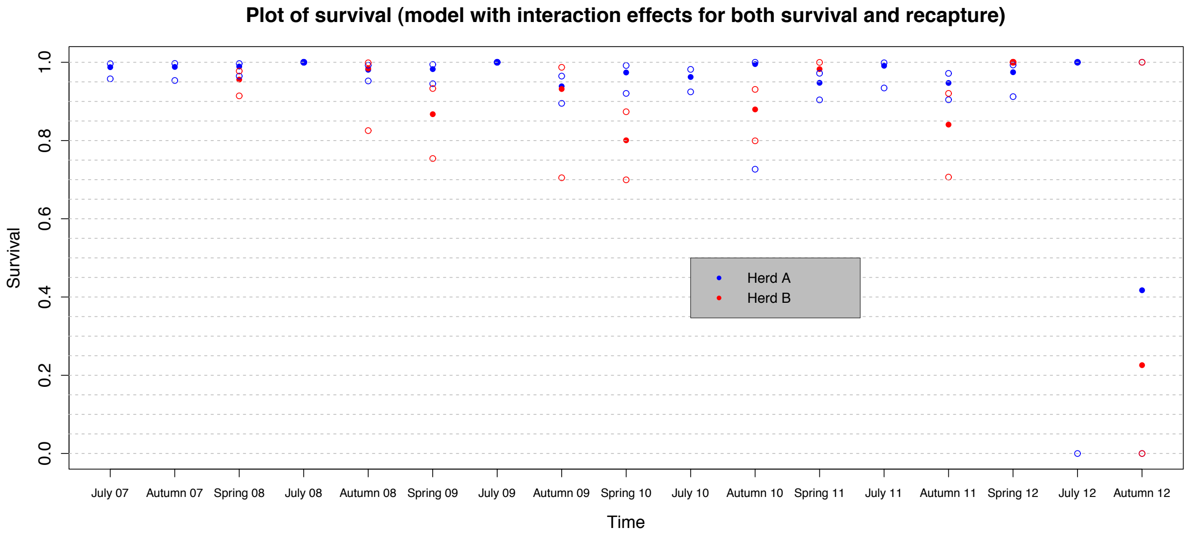

Fig S4.2. Survival estimates (filled circles) with lower and upper ends of 95% confidence intervals (open circles) for Model 2 in Table S4.1. Non-identifiable estimates have confidence intervals from 0 to 1.

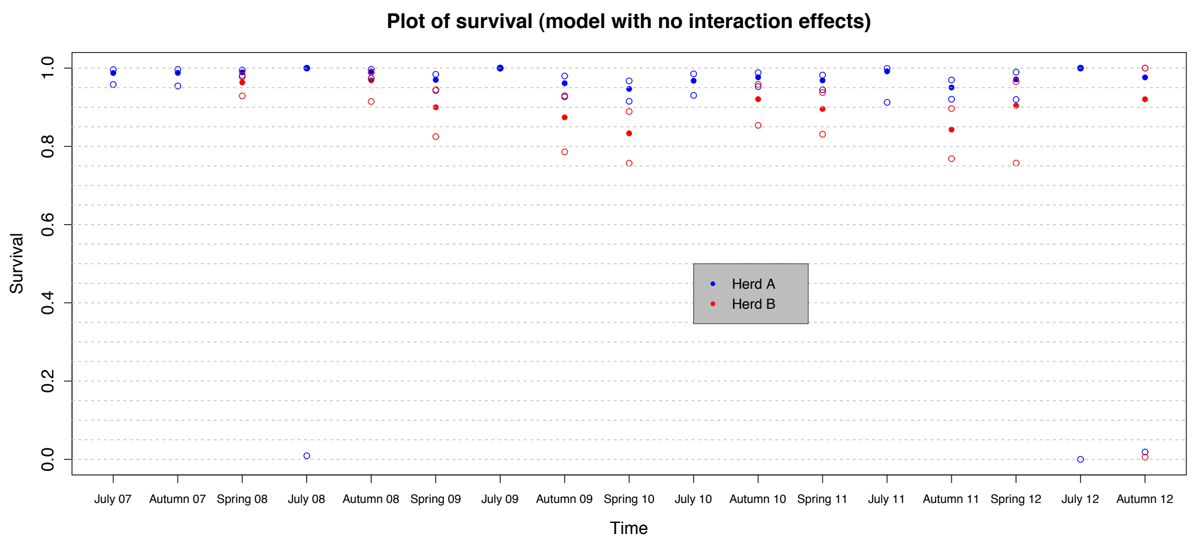

Fig S4.3. Survival estimates (filled circles) with lower and upper ends of 95% confidence intervals (open circles) for Model 3 in Table S4.1. Non-identifiable estimates have confidence intervals from 0 to 1.
