# Supplementary material for: High Female Mortality Resulting in Herd Collapse in Free-Ranging Domesticated Reindeer (Rangifer tarandus tarandus) in Sweden
Source: PLoS One. 2014 Oct 30;9(10):e111509. doi: 10.1371/journal.pone.0111509 (PMC4214728; doi:10.1371/journal.pone.0111509)
Supplement: Text S5 — Herd growth, recruitment and harvest. This text gives details on annual changes in herd size, recruitment of new calves and harvest from 1996 until 2001 for the whole (combined) herd, and from 2002 until 2011 for herd A and B separately. (PDF) [file pone.0111509.s005.pdf]

Text S5 - HERD GROWTH, RECRUITMENT AND HARVEST

Table S5.1. Annual herd growth (change in number of reindeer), growth rate (relative change), recruitment (calves per female in autumn before harvest), harvest (total number and per cent of available), and herd size after harvest

Year (autumn)	Herd growth*	Herd growth rate	Recruit-ment†	Harvest		Herd size after harvest
				number	% of available	
Whole herd (A+B)						
1995						1509
1996	910	0.60	0.57	572	24%	1847
1997	549	0.30	0.56	501	21%	1895
1998	613	0.32	0.62	593	24%	1915
1999	359	0.19	0.53	524	23%	1750
2000	1008	0.58	0.56	715	26%	2043
2001	279	0.14	0.60	649	28%	1673
Herd A						
2001						730
2002	278	0.38	0.54	247	25%	761
2003	479	0.63	0.53	315	25%	925
2004	88	0.10	0.62	127	13%	886
2005	460	0.52	0.44	265	20%	1081
2006	-81	-0.07	0.52	223	22%	777
2007	529	0.68	0.43	247	19%	1059
2008	191	0.18	0.35	110	9%	1140
2009	215	0.19	0.49	245	18%	1110
2010	430	0.39	0.46	288	19%	1252
2011	385	0.31	0.75	440	27%	1197
Herd B						
2001						943
2002	314	0.33	0.57	396	32%	861
2003	427	0.50	0.46	273	21%	1015
2004	-69	-0.07	0.60	73	8%	873
2005	471	0.54	0.39	181	13%	1163
2006	-295	-0.25	0.49	196	23%	672
2007	399	0.59	0.41	142	13%	929
2008	-19	-0.02	0.32	54	6%	856
2009	-187	-0.22	0.57	138	21%	531
2010	47	0.09	0.43	67	12%	511
2011	267	0.52	0.50	65	8%	713

*Change since the previous autumn
†Calves per female >1year old

The year to year variation in herd growth and herd size after harvest is based on annual reindeer counts and does not necessarily reflect true changes, but may partly be due to varying success in gathering and counting the herd.
